# Performance of the beta-glucan test and a dynamic prediction rule to identify patients in the ICU at high risk to develop candidemia

**DOI:** 10.1186/cc14149

**Published:** 2015-03-16

**Authors:** SA Nouer, AL Colombo, P Esteves, T Guimaraes, F Scapinello, B Grassi de Miranda, F Queiroz-Telles, M Nucci

**Affiliations:** 1 Hospital Universitário Clementino Fraga Filho, Rio de Janeiro, Brazil; 2 Federal University of São Paulo, Brazil; 3 Hospital Servidor Publico Estadual de São Paulo, Brazil; 4 Federal University of Parana, Curitiba, Brazil

## Introduction

Early initiation of antifungal therapy (AFT) improves the outcome in candidemic patients, but empiric AFT is not considered the standard of care.

## Methods

We used a scoring system based on the presence of a central venous catheter and receipt of antibiotics, plus at least two of the following: dialysis, surgery, pancreatitis, and receipt of corticosteroids, other immunosuppressive agents or parenteral nutrition. Different from the original description of the score which considered only the first 7 days of ICU stay, we selected patients who fulfilled these criteria at any time during the ICU stay. Once a patient fulfilled these criteria, AFT (anidulafungin 200 mg followed by 100 mg daily) was initiated provided that the patients also presented with any of the following: fever, hypothermia, hypotension, leukocytosis, acidosis or elevated C-reactive protein. Blood cultures (days 1 to 2) and baseline serum BDG (days 1 to 3) were performed. Patients with candidemia were treated for ≥14 days, those without candidemia but ≥1 positive BDG (≥80 pg/ml) received AFT for ≥10 days, and patients with negative blood cultures and negative BDG discontinued anidulafungin.

## Results

A total of 2,148 patients were screened, and 85 (4%) fulfilled entry criteria. The incidence of candidemia in these 85 patients was 8.2%, compared with 0.5% in the remaining 2,063 patients (relative risk 16.9%, 95% confidence interval (CI) = 6.63 to 43.55). Baseline BDG was positive in 74 patients (87%), with a median number of positive tests of 3 (range 1 to 3) and a median value of 523 pg/ml (range 83 to 6,860). All seven patients with candidemia had positive baseline BDG (median value 523 pg/ml, range 203 to 3,660). The best cutoff of baseline BDG for the diagnosis of candidemia was 522 pg/ml (area under the ROC curve 0.883, 95% CI = 0.769 to 0.997), with sensitivity and specificity of 86% and 88%, respectively. The cutoff value of 80 pg/ml had sensitivity and specificity of 73% and 27%, respectively.

## Conclusion

This dynamic prediction rule was able to differentiate a group of ICU patients at high risk to develop candidemia, with a relative risk of 16.9. BDG is frequently positive in ICU patients. A cutoff value of 522 pg/ml was able to discriminate between candidemic and noncandidemic patients. A revision of the cutoff value for BDG in the ICU is needed.

